# Protocol vulnerability detection based on network traffic analysis and binary reverse engineering

**DOI:** 10.1371/journal.pone.0186188

**Published:** 2017-10-19

**Authors:** Shameng Wen, Qingkun Meng, Chao Feng, Chaojing Tang

**Affiliations:** College of Electronic Science and Engineering, National University of Defense Technology, Changsha, China; Zhejiang University, CHINA

## Abstract

Network protocol vulnerability detection plays an important role in many domains, including protocol security analysis, application security, and network intrusion detection. In this study, by analyzing the general fuzzing method of network protocols, we propose a novel approach that combines network traffic analysis with the binary reverse engineering method. For network traffic analysis, the block-based protocol description language is introduced to construct test scripts, while the binary reverse engineering method employs the genetic algorithm with a fitness function designed to focus on code coverage. This combination leads to a substantial improvement in fuzz testing for network protocols. We build a prototype system and use it to test several real-world network protocol implementations. The experimental results show that the proposed approach detects vulnerabilities more efficiently and effectively than general fuzzing methods such as SPIKE.

## Introduction

As the use of complex and important network applications increases, network protocol security requirements become ever more significant. However, finding effective approaches for testing network protocol security has proven to be a difficult problem [1–3].

Fuzz testing is one important network protocol security test method. Fuzz testing involves injecting large amounts of data to test the security of applications, and it can also be used to detect vulnerabilities in network protocol implementations. In this paper, the main research objective is the application layer protocol, which includes the public protocols of both standard networks and private networks without the details. In some cases, we must have a deep understanding of the protocol format and protocol interaction process to make fuzz testing reach the deeper protocol states efficiently. The network traffic analysis based on block-based protocol description language can closely mimic the protocol to assist in generating suitable test cases. The binary reverse engineering method, which is based on the genetic algorithm (GA) and a fitness function, is designed to focus on high code coverage that can reach more vulnerable points.

In this paper, we introduce a novel method that combines network traffic analysis and binary reverse engineering to improve network protocol fuzz testing. Briefly, this work presents the following main contributions:

- A novel method is proposed that uses the block-based protocol description language for protocol format analysis and the GA focuses on high code coverage test packets.
- We also built a prototype fuzz testing system used to detect vulnerabilities in network protocol implementations.

## Related work

Reverse engineering research in the network protocol field tends to take one of two main approaches: one uses the network traffic to infer the network protocol, while the other dynamically tracks and analyzes executable programs using the network protocol. This latter approach is called binary reverse engineering or tainted data analysis.

Marshall Beddoe originated the practice of network traffic analysis in 2004 when he launched the Project Informatics (PI) project, whose goal was to find an algorithm to generate amino acids from DNA through biological analogy. Corrado Leita, Ken Mermoud, and Marc Dacier proposed using the PI project to automatically generate Honeyd configuration scripts [4]. Cui Weidong and Vern Paxson et al. proposed a solution for network protocol identification and automatic recovery called RolePlayer [5]. The idea is that, by obtaining the user input parameters, an interactive script can be generated based on a small amount of data from a sample stream. Subsequently, the system can identify whether a new section of the flow conforms to the learned protocol or not. This approach to network traffic analysis can universally adapt to various network protocols, including both public and private protocols.

The binary reverse engineering method can comprehensively track the process of network applications and is relatively accurate, but its implementation is complex and each protocol operating environment has special requirements. Protocol identification and fuzzing methods that involve binary reverse engineering are based on dynamic taint analysis. In recent years, dynamic taint analysis [6] has been used at the binary code level to achieve widespread tracking and analysis of untrusted data. Argos [7] and TaintCheck [8] are two typical examples. The dynamic taint analysis method is based on tainted data. In dynamic taint analysis, all network interactive data is considered to be from an untrusted data source. This method monitors process structural information to obtain the processes’ protocol formats. Caballero Juan proposed the Polyglot system based on this idea [9]. He considered network packets to be tainted input data, performed monitoring at the instruction level and extracted the semantic features. Based on the results of analyzing several monitored processes, the fused semantic information from all messages could be represented using the same format; thus, he was able to extract a universal protocol format. G. Wondracek adopted this method to identify single message structures; however, he extended the approach to identify multiple messages using a sequence alignment method [10]. Nevertheless, the approach to analyzing tainted data has some limitations. For example, taint analysis needs to operate a server program in a specified environment. In addition, tracking complex applications requires analyzing large amounts of data, which reduces server performance. The method in this paper combines the advantages of network traffic analysis and binary reverse engineering to improve the efficiency of network protocol vulnerability detection.

## Issue analysis

The key issue of vulnerability detection based on network protocol formats is how to construct the test data packets. The traditional black box testing techniques are realized on a client and based on a network protocol. Then, experts test the server based on their experiences of the causes of data overflow by constructing test packets and sending them to the target system. However, this approach has the following obvious defects.

1. Low test case hit rate. At present, the most serious problem with the traditional fuzzing method is that the test case hit rate is too low, especially for network service software. Because it lacks control over the target protocol, the server may directly refuse a test case sent by the fuzzing device or can break the connection prematurely. Fuzz testing of an open protocol is relatively simple because test cases can refer to detailed RFC and other public documents. Consequently, for open protocols, generating a series of effective, complete test cases is relatively simple. However, even when a protocol is both common and open, there is no guarantee that the application developers will comply strictly with the published standards. Moreover, fuzz testing a private third-party protocol, regardless of its simplicity, is also a challenge. Some subtle analyses can be achieved through reverse engineering, but only at a high computational cost. Therefore, building automatic protocol analysis methods is currently a significant problem [11].
2. Unable to determine when fuzz testing is complete. The number of test cases that can be generated based on variations of the fuzzing method are infinite; thus, determining the specific duration that the fuzzing method should operate is problematic. When all the current test cases have been generated, what should the fuzzing method do? When no abnormal trigger exists, it either repeats the current processing step or alters the generative rules. At present there is no clear standard for measuring and evaluating fuzz testing completion.
3. Locating the position of vulnerabilities is difficult. Often, several different test cases may trigger the same exception during fuzz testing. When an exception is triggered, the location of the abnormal exception handler is generally far away from the true anomaly, which leads to problems in identifying the real vulnerability.
4. Multi-dimensional fuzz testing problems are not considered. At present the majority of test case generation approaches based on fuzz testing are one-dimensional, namely, they generate a single mutation on one input element one time. However, triggering many vulnerabilities requires multiple elements working together. Testing such vulnerabilities requires a multi-dimensional fuzz technique. Currently, this advanced approach has several problems, including combinatorial explosions, insufficient vulnerability coverage, and so on.

To address these defects in traditional vulnerability detection approaches, we used the proposed method in this paper, which combines network traffic analysis and binary reverse engineering, to generate test cases. This approach better conforms to the protocol format, improves the test case pass rate, and can reveal network protocol vulnerabilities more effectively and efficiently.

## Framework design

In this paper, the design and implementation of the protocol vulnerability detection system are divided into three main parts.

**Fig 1:**
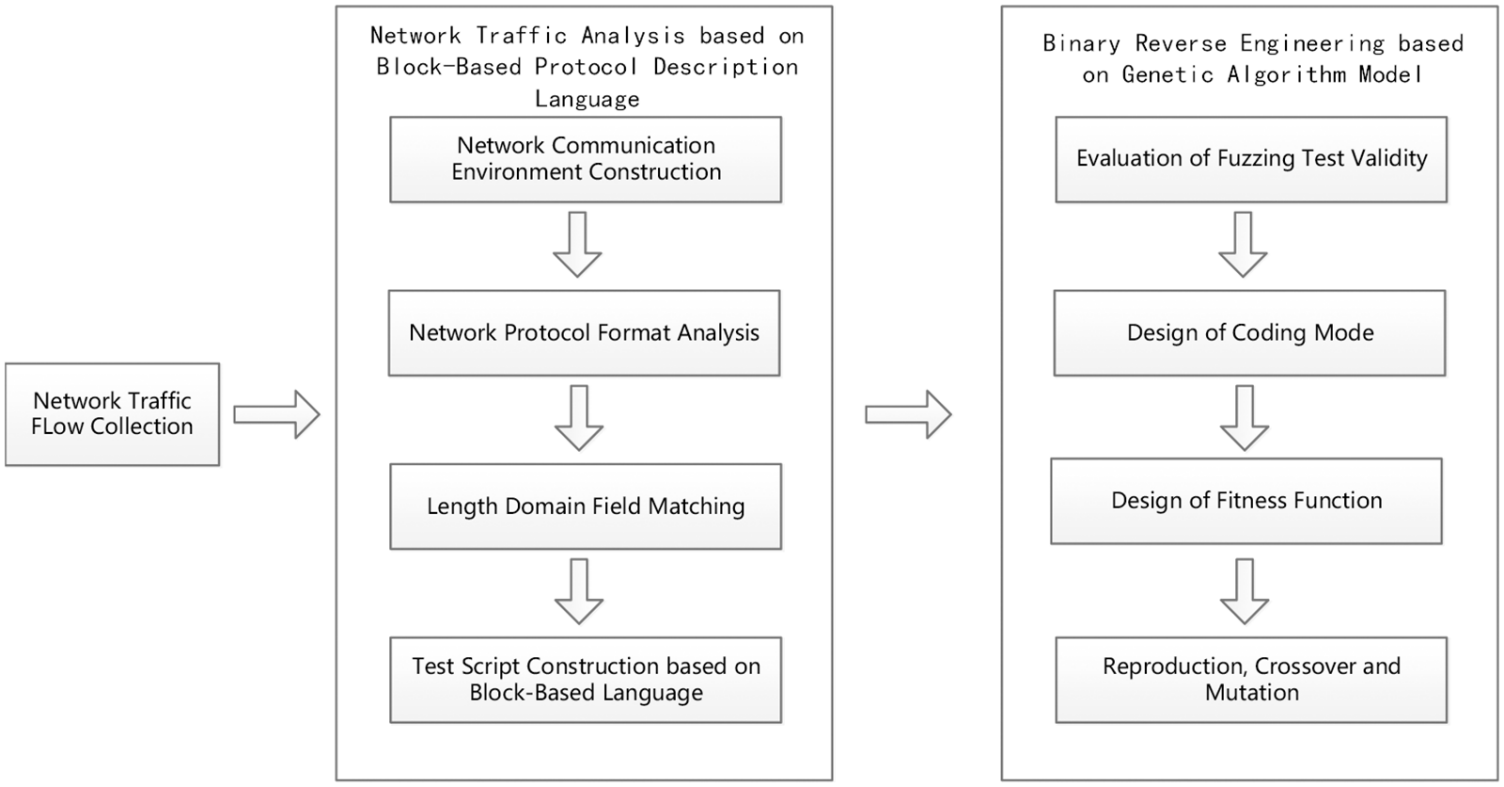
Framework of the proposed method.

First, in a preprocessing phase, we manually collect target network traffic flow packets, which form the information source for the subsequent analysis. The next phase is network traffic analysis, which is based on the block-based protocol description language. By constructing a network communication environment, the network protocol format is analyzed through fuzzing. The key problem involves matching the length domain field. In this study, Wireshark is used to parse the protocol format, and the block-based protocol description language is utilized for test script construction. The third phase involves applying reverse engineering technology. Here we analyze message information to generate vulnerability detection fuzzing test cases. Based on an evaluation of fuzz testing validity, we propose code coverage as a metric. In addition, we introduce the GA model to produce better test cases through a designed coding mode and fitness function. The algorithm then evolves test cases through reproduction, crossover and mutation. The framework of the proposed approach is shown in Fig 1.

## Network traffic analysis based on block-based protocol description language

Network traffic analysis based on the block-based protocol description language involves four procedures: constructing the network communication environment, analyzing the network protocol format, matching the length domain field, and constructing test scripts using the block-based protocol description language.

### Network communication environment construction

**Fig 2:**
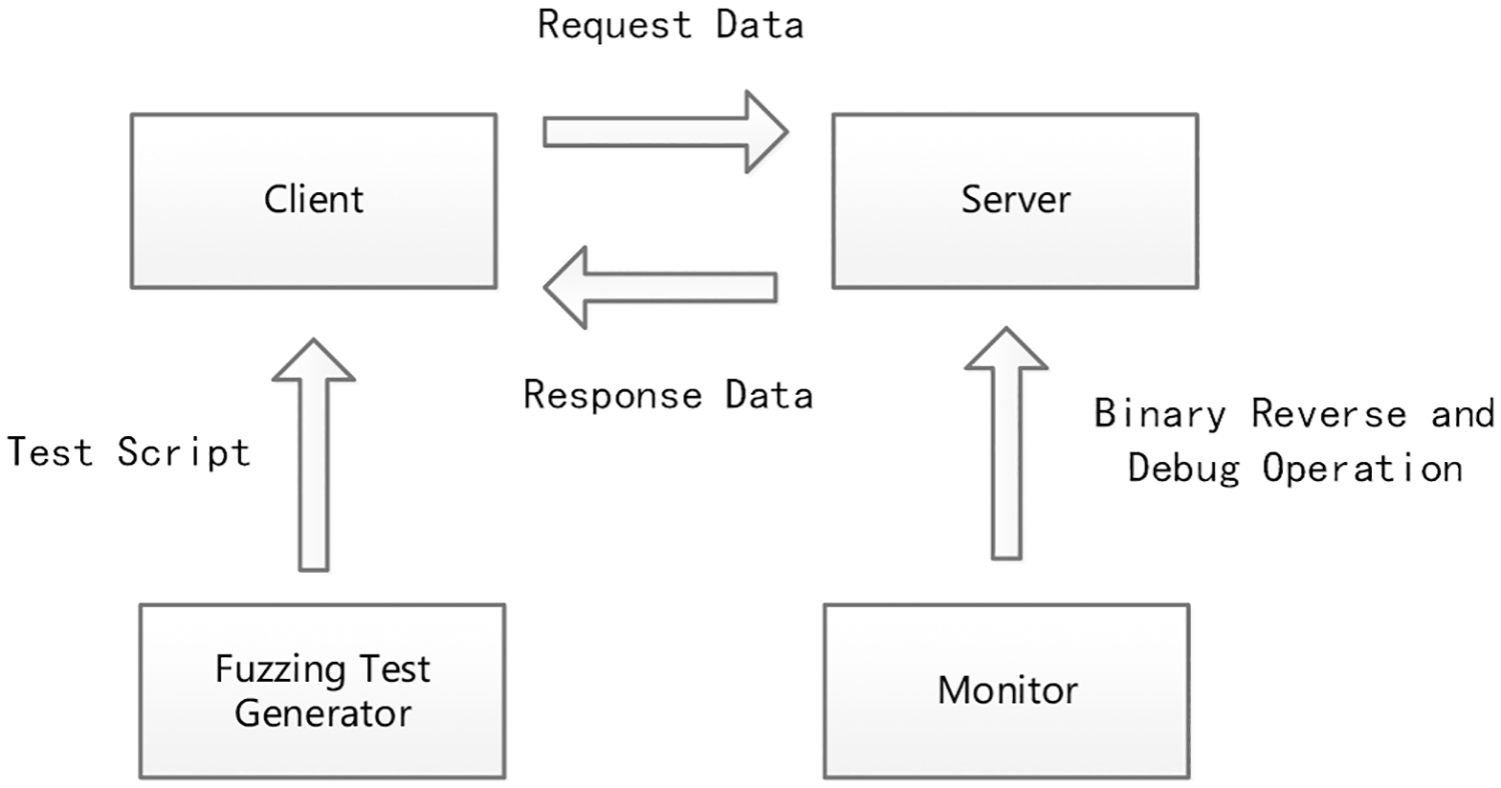
Vulnerability detection in C-S mode.

In essence, network communication involves client-server (C-S) mode, as shown in Fig 2. In the TCP/IP communication protocol model, a client sends a request data packet and the server returns a response data packet, called request-response mode. Therefore, network communication consists of the interaction of data packets. Each network service has a corresponding application layer protocol, which determines how the server interprets the contents of request packets. The details of the protocol are embodied in the data packets. When using client-server mode during vulnerability detection, a fuzz testing generator constructs test cases using a test script and sends them to server. Simultaneously, a running monitor is used to catch server exceptions.

### Network protocol format analysis

**Fig 3:**
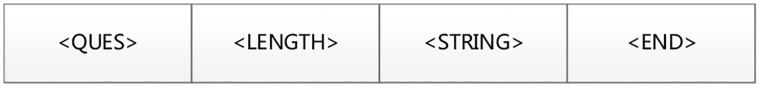
Protocol format analysis model.

Understanding the format of the protocol being tested is extremely important in researching vulnerability detection based on network protocols. In this paper, the network protocol format is abstracted to the model shown in Fig 3.

The variables in the protocol format are expressed as follows.

**QUES**: The format of the data packet; each protocol has a different format. This is a fixed value.

**LENGTH**: The length of the variable STRING that follows the header. This is a variable value.

**STRING**: The data section, which can contain any content. This is a variable value.

**END**: The format of the data packet. This is a fixed value.

It has been found that traditional black box testing techniques cannot achieve high hit rates when the protocol format is unknown. If the network protocol is jointly determined by the client and server API, or when testers have access to the client source code, then these APIs and the client source code can differ from the test assumptions and affect the test results, resulting in great differences between the expected and actual test results.

Even if the test has comprehensive knowledge of a known network protocol, it is still quite difficult to construct a client test program based on that network protocol. Moreover, test programs for other protocols may be unavailable, which wastes resources.

Fig 3 shows that the fixed values **QUES** and **END** in the packets must be known. Thus legitimate test packets can be created only when the protocol format is well understood. The proposed method is quite different from the random fuzzing technology and can achieve better results.

### Length domain field matching

Traditional black box tests are designed using the Perl scripting language to generate network data. This approach often replaces characters with long strings to find buffer overflow vulnerability. But when this technique is extended to more complex protocols, such as protocols formed of several layers (for example, the OSI seven-layer protocol model), this method is invalid. In a multilayer protocol, the sizes of each layer are associated, with the objective of preventing simple network data package imitations. In fact, most of today’s protocols are dependent on other protocols, such as HTTP.

Any protocol can be divided into a length domain and a data domain. However, for test subjects to construct their scripts, it is necessary to know the lengths of all the higher-level protocols to construct the underlying data package. If the relationships between the layers are not addressed well, the data packets will be rejected by the server.

For example, when a test tries to send an extra-long string to a particular application, replacing a character with an extra-long string is not easy. The tester must also dynamically update the length field in the HTTP header. Moreover, more complex protocols include more length fields that need to be updated. Thus, the traditional fuzzing method that sends random data packets will simply waste time due to mismatched network packets.

However, construction a function to calculate the length of each string for various protocols is difficult, and the reusability of such a function is not high. As a result, a technical framework is needed to isolate the underlying protocol and the lengths of the known high level protocols. For the artificial simulation protocol format, the best way is to consider the protocol as a long string of characters rather than as a multilayer network protocol model. The block-based protocol description language allows a tester to create many data blocks and bind them to the lengths of each domain. In this way, after the data block size is changed by replacing a shorter string with a longer one, the tester could recalculate the data block size accurately and send a request containing the correct length value.

### Test script construction based on block-based language

The block-based protocol description language is used to solve the dynamic matching problem of the length domain field, which can reduce the size of the fuzz input space significantly. Generally, protocol analysis methods grab a sample package and then parse the protocol format.

Automated capture tools are used to grab sample packages from the network. In this paper, Wireshark 2.0.2.0 is used as the protocol grabbing and recognition engine. Wireshark can identify more than 935 different protocol formats.

Based on an understanding of the protocol format, the next step is to write test scripts. Typically, manually written scripts often generate errors. Traditional vulnerability detection techniques show that writing test scripts is the most time-consuming portion of the task. Wireshark uses a PDML file as the tool to achieve test script automation.

To perform vulnerability detection more efficiently, protocol data packet construction is vital. Good test cases should conform to the network protocol, yet be able to contain deformed data packets. In this case, a deformity means the possibility that a packet will cause a vulnerability. To do this, test script construction based on the block-based protocol description language is introduced to specify the method and content of test data packages.

In the block-based protocol description language, a protocol format is divided into a length domain field and a data domain field. For manual analysis, a network protocol can be considered as a long string of characters rather than as the traditional multi-layer protocol model. The main functions available in the block-based protocol description language are listed in Table 1.

**Table 1:** Main functions in the block-based protocol description language.

No	Function	Description
1	string(“dummy”)	Define a constant character
2	string_uni(“dummy”)	Define a constant character width character
3	send(“block”)	Send data block
4	recv(“block”)	Receive data block
5	fuzz_ string(“dummy”)	Transform fuzz character to dummy
6	fuzz_ string_uni(“dummy”)	Transform fuzz width character to dummy
7	fuzz_ hex(0xff ff \xff)	Return hexadecimal value of fuzz
8	block_ size_ b32(“block”)	Define a 32-bit big-endian data block
9	block_ size_ l32(“block”)	Define a 32-bit little-endian data block
10	block_ size_ b16(“block”)	Define a 16-bit big-endian data block
11	block_ size_ l16(“block”)	Define a 16-bit little-endian data block
12	block_ size_ 8(“block”)	Define an 8-bit data block
13	block_ size_ hex_ string(“block”)	Define a data block of hexadecimal size
14	block_ size_ dec_ string(“block”)	Define a data block of decimal size
15	hex(0x0a 0a \x0a)	Define a hexadecimal constant character
16	block_ begin(“block”)	Define the start point of a block
17	block_ end(“block”)	Define the end point of a block
18	block_ crc32_ b(“block”)	Define crc32 in a big-endian data block
19	block_ crc32_ l(“block”)	Define crc32 in a little-endian data block
20	hex(0x0a 0a \x0a)	Define a hexadecimal constant character

Test script construction is an important component of vulnerability detection. Using the block-based protocol description language, the content that conforms to a protocol can be easily constructed. The key procedure is the conversion of PDML files using the block-based language.

The function of PD2AD [12] is to convert the PDML files, which contain protocol packets captured by Wireshark, into AD format. As long as the protocol is supported by Wireshark, the PDML file format can be converted successfully. For protocols that Wireshark does not recognize, we can still use the PDML file to parse the protocol by calling a custom function. The major structural morphology is shown in Table 2.

**Table 2:** Design of structural morphology.

//define major structural morphology typedef struct configuration { **char** *xml_filename; //point to XML-PDML file pointer **int** invert; //define packet packet > send = 0; second packet > send=1 **unsigned int** packet_counter; //number of packet grabbed **unsigned int** protco_counter; //number of protocol type **unsigned int** transport_type; //type of transform protocol:tcp=1;udp=2; **unsigned int** ip_clinet; //ip address of the client **unsigned short** port_server; //port of the client **unsigned int** ip_server; //ip address of the server **unsigned short** port_server; //port number of the sever **unsigned int** ip_pkt; //ip address of the current packet **unsigned short** port_pkt; //port number of the current packet **unsigned int** send; //send=1 means send data, else means receive data xmlDocPtr doc; //including tree stuctral pointer XmlNodePtr cur; //point to single node pointer … } config;

## Binary reverse engineering based on the GA model

Binary reverse engineering based on the GA model includes four procedures: evaluation of fuzz testing validity, design of coding mode, design of fitness function and genetic operations. An evolutionary fuzz testing method is designed to focus on high code coverage. In the proposed method, functions and basic blocks are identified using static analysis tools. Next, we set a break point on the corresponding function or basic block. Then, seed test cases are produced using the GA model. The fitness function selects the best test cases, and the next test set generation is constructed using reproduction, crossover and mutation.

### Evaluation of fuzz testing validity

The key issue in evaluating the validity of fuzz testing is how to measure the adaptability of the test data. Here, we consider the code coverage that can be achieved as the metric. Code coverage is a measure used to determine how much code has been executed and can be applied to both source code and binary files, although it is usually applied to source code. Code coverage is an important indicator that can both reflect test case coverage and measure test progression. Based on testing requirements, code coverage can be subdivided into: statement coverage, decision coverage, condition coverage, path coverage, and so on.

Code coverage is a very important index in the process of fuzz testing, which can help to better carry out fuzz testing. First code coverage rate is beneficial to evaluate the fuzzing overall test completion, it shows what code need to focus and guides the generation of new test cases to help find more potential vulnerability; on the other hand, a lot of repeated operations could be avoided by analyzing the code coverage to improve the efficiency of fuzz testing. When analyzing code coverage, it can be found that many fuzzing template tests, such as for database system function test cases, almost covered the same code block, so as long as the execution of a test case is equivalent to the execution of all the cases, which can significantly improve the efficiency of fuzz testing.

It is both time consuming and unnecessary to step through a complete program to evaluate code coverage. Instead, there are two main methods for evaluating code coverage. First, considering how functions are implemented, each function will have an entry point, and generally has a return point (unless a fatal exception occurs during function execution). Consequently, code coverage can be evaluated by tracing the function calls. Second, we also take basic blocks into consideration for code coverage. A basic block is defined as a sequence of instructions that are guaranteed to execute in sequence. By tracking the implementation of basic blocks (which involves tracking instruction execution), code coverage can be calculated accurately.

### The GA model

The GA [13] is based on ideas taken from natural selection. Through the design of a fitness function and the processes of genetic variation, the algorithm is intended to find a global optimal solution using an adaptive search method. In the GA, the concept of the survival of the fittest from natural selection is important and is translated to some index that represents a selection mechanism for each population generation. The GA has unique advantages in solving nonlinear search problems. The GA generally follows this process.

First, an initial population is generated. In this process, we need to encode groups based on specific issues to better adapt to different search conditions. Then, the GA uses a fitness function to calculate the fitness of each chromosome. The fitness function is defined by the user of the algorithm based on problem-oriented selection criteria. After calculating the fitness function value of each chromosome, three genetic operators named reproduction, crossover and mutation are applied, a new population is generated to simulate the survival of the fittest automatically. This process continues until a termination condition is reached or the algorithm has been repeated N times. At that point, the GA terminates, yielding the optimal solution.

### Design of coding mode

The key to evolutionary fuzz testing using the GA is to transform the problem of test case optimization and automation for protocol analysis by using the GA to find optimal solutions. The first step in this transformation is to convert the parameters of the actual problem space to a representative individual, which is composed of genes. This process is called coding, and it is a data representation of the test cases.

To meet the GA’s requirements for crossover and mutation operations, each test case must be expressed as a combination of genes. During the process of evolution, if the test case is composed of a single gene, the protocol constraints between individual elements can easily be destroyed. Thus, the coding mode is slightly more complex than a single gene structure.

An interaction between a client and a server is called a session, during which a data packet is sent to the target application. Each session consists of a number of legs, and each leg contains numerous tagged nodes named tokens. Each token is a single data item that can include both data type fields or data content fields such as field data length, string type data, binary type data, ans so on. To avoid premature convergence of the simple GA, a nested structure called a session pool is used in practical coding implementations. A session pool consists of several ordered sessions. The size of session pool can be set by the max number of session, the max number of legs and the max token of each leg. The initialization is based on seed or random.

For convenience and simplicity, we choose the binary coding mode. In this mode, every element of the parameter vector is coded as a string consisting of zeros and ones.

### Design of fitness function

A fitness function is a measurement standard applied to the test cases in the GA model. The GA does not use external information during the process of evolution; instead, only the fitness function is used to search the population. Thus, the fitness function determines the evolutionary direction of the population. When the fitness function is improperly designed, some extraordinary individuals will occur at the early stages of the genetic model, which affects the global optimization performance.

In fuzz testing, a good test case is a combination of data that can trigger a program exception. The goal of evolutionary fuzz testing is to analyze the protocol automatically. Therefore, good test cases should be designed in accordance with the protocol provisions. When more of the test data conforms to the protocol format, the possibilities for processing the test cases through the network increase, potentially triggering more vulnerabilities.

In this paper, the target binary function and basic block entry address are extracted with static analysis. Then we set a breakpoint on the target function or basic block entry address using a debugger. During fuzz testing, we record coverage during execution of the predefined breakpoint test cases, and calculate the code coverage using the hit rate of the predefined breakpoints. The fitness function designed not only considers the hits on the self-session pool but also their affect on all session pools.

### Reproduction, crossover and mutation

Reproduction is usually the first operator applied to a population. The reproduction operator is intended to make the chromosomes with higher fitness values survive at a higher probability. The roulette-wheel selection method [14] is usually chosen as the realization principle of the reproduction operator.

**Fig 4:**
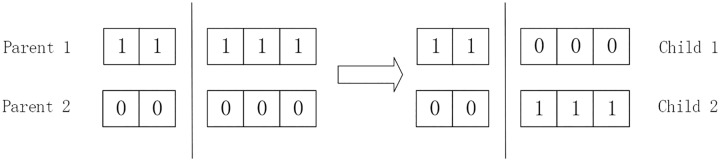
An example of crossover operator.

The role of the crossover operator is to mix the contents of a pair of chromosomes to improve population diversity. In a single-point crossover operator, the two strings are cat at an arbitrary place and the same one portion of these two strings are swapped with each other to create two new strings. An example of crossover operator is shown in Fig 4. The crossover rate controls the frequency of the crossover operator. With a higher crossover rate, the new structures are introduced into the population more quickly. However, too high crossover rate causes high-performance structures are discarded faster than selection can produce improvements. Too low crossover rate causes the search gets stuck with the lower exploration rate.

**Fig 5:**
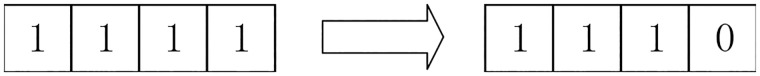
An example of mutation operator.

The role of the mutation operator is to modify the values of some encoded chromosomes. Mutation helps prevent the search process from falling into a local maximum, but a mutation rate that is too high can lead to huge fluctuations. The mutation operator changes a 0 to a 1 and vice versa with a small mutation probability. An example of mutation operator is shown in Fig 5.

## Experiments and results

In this section, we use the proposed method to test FTP and TFTP protocols. The experiment involves a QEMU virtual platform with a host machine and a guest machine. The host machine contains an Intel^®^ Core^™^ i7-6700K CPU running at 4 GHz with16 GB memory and the Ubuntu 14.04 operating system. The guest machines are running Linux Debian 7 and Windows XP SP3 operating systems. The target programs execute in the guest machine. The prototype fuzzing system and the general fuzzing tool SPIKE (S1 File) execute on the host machine.

### FTP protocol test

The File Transfer Protocol (FTP) is a standard network protocol used to transfer files between a client and server on a computer network. The FTP protocol establishes a control connection and a data connection and has two different modes: PASV and PORT. Our experiment uses the PORT method as the test mode.

Usually, the protocol analysis method uses packet capture tools to collect data packets during communications between the client and the server. In this experiment, Wireshark version 2.0.2.0 is selected as the packet capture tool. To date, Wireshark can identify more than 930 protocol formats. It saves captured packets as PDML files. Based on the syntax parsing and block-based protocol description language, we can easily convert files in PDML format to a related test script. Some partial test script results are shown in Table 3.

**Table 3:** Description of FTP protocol based on block-based language.

//description of tcp FTP protocol based on block-based language block_begin(“packet_1”); //fixed value string(“QUES”); //fixed value block_size_b32(“string_1”); //big endian 32 bits size fuzz_string(“***”); //fuzzing data block_end(“string_1”); string(“END”); string(“QUES”); //fixed value block_size_b32(“string_2”); //big endian 32 bits size fuzz_string(“***”); //fuzzing data block_end(“string_2”); fuzz_string(“END”); hex(0a); //\n … block_end(“packet_1”); send(“packet_1”); //tcp …

We chose seven real-world FTP programs as targets to test several known vulnerabilities. These programs are Freefloat FTP Server, Core FTP, PCMan FTP Server, SurgeFtp Server, Konica Minolta FTP Utility, and KnFTPd (S2 File). Through pre-analysis, we found that these FTP programs have many functions. We removed some functions, including startup, shutdown, configuration files operations, and others. Instead, our experiments focus on dangerous functions that are directly related to the attack surface, which comprise approximately 10% of the total.

To compare the effects of vulnerability detection, we compared our method with SPIKE [15] based on the experimental objective using the general fuzz method. A comparison of the results of the proposed method and SPIKE for vulnerability detection is shown in Table 4. The experimental results validate that our system can successfully detect known vulnerabilities more effectively than SPIKE.

**Table 4:** Comparison of the proposed method and SPIKE in vulnerability detection using applications running the FTP protocol(S: Success, F: Failure).

Software Name	Software Version	Vulnerability Number	Vulnerability Type	The Proposed Method	SPIKE
Freefloat FTP Server	1.0	CVE-2012-5106	Buffer Overflow	S	S
Core FTP	2.1 build 1612	CVE-2009-3484	Buffer Overflow	S	S
PCMan FTP Server	2.0.7	CVE-2015-7601 CVE-2013-4730	Directory Traversal Buffer Overflow	F S	F F
SurgeFtp Server	2.3a1	CVE-2013-4742	Buffer Overflow	S	S
Konica Minolta FTP Utility	1.0	CVE-2015-7767 CVE-2015-7768	Buffer Overflow Buffer Overflow	S S	S S
KnFTPd	1.0.0	CVE-2012-5905	Buffer Overflow	S	S
Detection Rate				87.5%	75%

### TFTP protocol test

TFTP stands for Trivial File Transfer Protocol. TFTP is a simplified version of the FTP protocol and is also used for transferring files between network devices.

We choose six real-world TFTP programs as the targets to test several known vulnerabilities. The targets are Tftpd32, 3Com 3CTftpSvc, Cisco Tftp Server and Serva32 (S3 File).

For this experiment, we also selected SPIKE as the comparison method. Table 5 shows a comparison of the results of the proposed method and SPIKE in vulnerability detection. Again, the results validate that our system is more effective than SPIKE in detecting known vulnerabilities.

**Table 5:** Comparison of the proposed method and SPIKE in vulnerability detection with TFTP protocol programs.

Software Name	Software Version	Vulnerability Number	Vulnerability Type	The Proposed Method	SPIKE
Tftpd32	3.51.0.0 3.0.1	CVE-2013-6809 CVE-2006-6141	Format String Buffer Overflow	S S	S S
3Com 3CTftpSvc	2.0.1r	CVE-2006-6183	Buffer Overflow	S	S
Cisco Tftp Server	1.1	CVE-2010-1174	Denial of Service	F	F
Serva32	2.1.0	CVE-2013-0145	Buffer Overflow	S	F
Detection Rate				80.0%	60.0%

## Conclusions

Fuzz testing technology is currently used widely for vulnerability detection. Its principles are simple and the process of finding vulnerabilities that can be reproduced is also convenient. However, because of the complexity of the targets, fuzz testing also has some defects and limitations, particularly in detecting vulnerabilities in network protocols.

This paper presents a novel method of fuzz testing that fully considers the characteristics of network protocol vulnerability detection by combining network traffic analysis and binary reverse engineering. The proposed method introduces the block-based protocol description language for protocol format analysis and uses the genetic algorithm to generate test cases that focus on code coverage. Through experiments, we show that our method is both effective and efficient.

In possible future work, we plan to use our method to test other complex network protocols and continue to strive to improve our method’s performance.

## Supporting information

S1 File SPIKE. (ZIP)

S2 File FTP programs. (ZIP)

S3 File TFTP programs. (ZIP)
