# Supplementary material for: Protocol vulnerability detection based on network traffic analysis and binary reverse engineering
Source: PLoS One. 2017 Oct 19;12(10):e0186188. doi: 10.1371/journal.pone.0186188 (PMC5648143; doi:10.1371/journal.pone.0186188)
Supplement: S1 File — (ZIP) [file pone.0186188.s001.zip › S1_file/SPIKE/SPIKE2.9.tgz!/SPIKE/SPIKE/documentation/advantages_of_block_based_analysis.pdf]

The Advantages of Block-Based Protocol Analysis for Security Testing

Dave Aitel
Immunity,Inc.
111 E. 7th St. Suite 64, NY NY 10009, USA
dave@immunitysec.com
February, 4 2002

Abstract. This paper describes a effective method for black-box testing of unknown or arbitrarily complex network protocols for common problems relating to the security of a program or system. By introducing a block-based method for taking advantage of all known factors in a network protocol, and delimiting the effect of all unknown factors, the potential space of inputs to a program can be reduced intelligently by a tester, compensating for incomplete knowledge of the target's implementation or design.

Introduction

Traditionally black-box testing of network protocols has been performed by creating a rough client of the network protocol to be tested and then manually permuting the protocol in ways the tester thinks will cause a software fault in the target system. This is inefficient in several ways:

- If the network protocol is defined by both server and client API's or the tester has source for the client, it is likely that these API's or source codes will influence the assumptions made by the tester, which will then be in line with the assumptions made by the developers, causing gaps in testing
- Even given complete knowledge of the protocol, creating a client for a protocol can be a large project, and that client is rarely portable to other protocols, even of a similar nature
- Many times testers have only limited knowledge of the protocols under attack or limited knowledge in the ways that the protocol may break

In addition, gray-box testing, where a program is instrumented and its data flow is analyzed while it is being stressed by network input, is difficult to both scale to arbitrarily complex systems and port to other architectures. In fact, setting up more than a minimal test frame for a program can change a test's results in ways that cloak vulnerabilities, by introducing latency or modification

of the program's internal memory state.

To address these weaknesses, the author developed and tested a framework for simulating network protocol clients and automating black-box testing based on that framework. This framework, implemented as a C API and a C-like scripting language, allows a black-box tester to leverage any existing knowledge of a protocol, or similar protocols, while testing. It also allows both manual and programmatic exploration of the potential space of network inputs, which gives the tester a more efficient way to find software faults, such as buffer overflows, integer overflows, or memory allocation errors.

The need for flattened protocol stacks for fuzzing

The history of black-box testing is strewn with examples of Perl scripts designed to replicate some form of network traffic, replacing a small string with a larger string in order to overflow a buffer. However, when attempting to expand this technique into a more complex application protocol, consisting of several layers, the sizes of each layer become correlated in away that precludes a simple modification of a network packet. In effect, today's network protocols depend themselves on other application layer protocols, such as HTTP.

Any protocol can be decomposed into length fields and data fields, but as the tester attempts to construct their test script, they find that knowing the length of all higher layer protocols is necessary before constructing the lower layer data packets. Failing to do so will result in lower layer protocols rejecting the test attempt.

As an example, if a tester wants to send a long string into a particular web application, it is not enough to simply modify an existing request (a POST) by replacing a variable with a longer string. The tester must also update the Content-Length: field in the HTTP header. For more complex protocols, there will be many length fields that need to be updated, some of them being calculations based on the lengths of the encapsulated protocols. Blind fuzzing, that is, sending purely random and unformatted data, is thus largely a waste of time.

However, creating a custom programmatic function to calculate each length is onerous. In addition, the creation of a functional or object oriented description of each protocol does not lead itself to reuse or manual modification. There is thus a need for a framework that isolates the layers below a protocol from having to know the components or component lengths of any of the upper layer protocols. For manual modification, it is best if the protocol is treated simply as a long string of bytes, rather than a nested egg-within-an-egg of protocols. The ability to flatten out a protocol stack in this way while

automatically calculating lengths is the main effect of the author's SPIKE framework.

Creation of the SPIKE Framework

The data structure that provides for block—based protocol modeling, known as a SPIKE, is a simple list of structures which contain block size information and a queue of bytes. Both binary data and size placeholders are pushed onto the queue. Whenever a size placeholder is pushed, a new block-size structure is allocated and given a unique name. For example, examine the simple SPIKE script below:

```
s_block_size_binary_bigendian_word(“somepacketdata”);
s_block_start(“somepacketdata”)
s_binary(“01020304”);
s_block_end(“somepacketdata”);
```

This script first pushes four null bytes onto the SPIKE's queue, but also allocates a block listener named “somepacketdata” and sets the internal state of that block listener to be a big endian word. The script then starts the block “somepacketdata” which searches all the available block listeners for any looking for the string “somepacketdata” and updates their internal “start” pointers. Then 4 bytes (0x01020304) are pushed onto the queue, and finally the block is ended, at which point any listeners' sizes are finalized, and the initial four nulls (a big endian word is four bytes long) are filled in with the proper value.

This SPIKE script snippet is somewhat simplistic, but the power of SPIKE directly derives from its ability to isolate the lower layer protocols (HTTP, or NetBIOS, for example) from the higher level protocols (such as XML-RPC or SMB). A SPIKE script, unlike a traditional fuzzing platform, does not need to pre-construct all higher-layer protocols in order to determine sizes. Instead, the size calculations themselves are deferred until blocks are closed. This prevents any one layer from having to know the internals of any other layer. Blocks can be nested or intertwined in any way, depending on the network protocol being modeled.

Fragmentation of packets is one area where this technique becomes inappropriate, and these cases are handled specially. But even in these cases, which are typically complex RPC protocols, a SPIKE is used to construct each fragment (a PDU in Microsoft RPC is a good example) and encapsulate arbitrary numbers of data types within the fragment.

Representing Data Types with SPIKE

Because most network protocols need to marshall and unmarshall the same kinds of data, they have evolved to do it in roughly the same ways. There are many examples of strings being marshalled with a size in big endian word format, then the ASCII string, then a null byte, then some pad data to make the string data structure aligned on a four-byte boundary. Each protocol designer typically uses their own code to encode this data type, but users of SPIKE use one generic function call, which may internally use block-based code to generate the data type, if the data type is complex.

As SPIKE matures, it's collection of encoding routines becomes more and more complete. A Unicode string sent over the wire by any of Microsoft's protocol implementations is sent in a similar format, so once the work done to create a valid SMB packet is done, the data types created work naturally with Microsoft's implementation of DCE-RPC.

Of course, even raw primitives are quite useful, such as creating a halfword in Intel-byte-order or big endian order (regardless of the SPIKE host's platform) or understanding different forms of hexadecimal formats ("0x0001" and "0001" and "00 01" are all parsed correctly by s_binary(), allowing a tester to cut-and-paste from almost any source.)

Automating Fuzzing

Once a protocol is represented in a linear state, certain parts within it are marked as variables. This is typically done by simply appending _variable() to the function name.

For example:

```
s_string_variable("POST");
s_binary_bigendian_word_variable(0x01);
s_unistring_variable("\\PIPE\\");
```

The SPIKE script fragment above marks each of these date types as a variable. Each SPIKE data structure keeps track of which variable it is now fuzzing. A simple loop iterating over the SPIKE allows it to change first the POST into a set of long strings, then the 0x01 into a set of integers known to trigger integer overflows, then the Unicode string into a set of long strings. Each "long" string is taken from a global set of strings known to cause problems in various protocols. As new classes of attack are discovered, strings are added to this global list.

It should be noted that size directives also can be used as integer variables. Because the protocol has been flattened out, there is a simple linear

progression through both the normal variables and size variables.

Measurable Results

It should be noted that SPIKE has been under development for over a year, and during that time has discovered literally dozens of new vulnerabilities, some of which have been DCE-RPC vulnerabilities. This is important in retrospect only because the efficacy of a block-based strategy for protocol modeling and fuzzing is hard to measure. It may be measurable only subjectively, in terms of the “ease-of-use” aspects for creating a new protocol modeler, and finding a new vulnerability. What few concrete measurements can be taken are the result of finding remote vulnerabilities in services which are known to be vulnerable, but for which the particulars of the vulnerabilities are unknown. The abilities of the fuzzing framework can then be tested as the time-spent, versus ability to discover the vulnerability. In many cases, more than one vulnerability is discovered.

For the purposes of this paper, a DCE-RPC over named pipes fuzzer was built into SPIKE version 2.8 to attempt to locate the RPC Locator exploit (described in MS-03-01). Building Netbios+SMB+DCE-RPC into SPIKE was simplified by the fact that SPIKE 2.7 already had incorporated some DCE-RPC functionality. The process of building a new protocol into SPIKE typically incorporates any knowledge about the protocol that can be gleaned from common protocol dissectors such as Ethereal, and in this case, both Netmon and Ethereal have quite full descriptions of SMB and DCE-RPC.

Nevertheless, it is SPIKE's unique block data structure that allowed a reasonably complete DCE-RPC over SMB over Netbios stack to be completed within one day, and debugged over the next few days. SPIKE's RPC fuzzers now include (as of version 2.8) a SunRPC fuzzer, a DCE-RPC fuzzer, and a DCE-RPC over named pipes fuzzer. Each of these works in a similar fashion: they bind to and then communicate with a remote RPC service, sending random numbers of random but valid data types as the parameters to a function call. These data types are chosen to both pass through any demarshalling routines on the remote end, and attempt to overflow the function handler.

For example, in hindsight, the Microsoft RPC Locator service will properly demarshall the following SPIKE code and overflow:

```
s_intelword(0x03); /*must be 0x03*/
s_intelword(rand()); /*can be anything*/
//next a long string in the form of “/.../<string>/”
s_msunistring(“/.../AAAAAAAAA......AAAA/”);
for (i=0; i<13; i++)
```

```
  s_intelword(0x00);
/* the final padding words can be anything, but zeros
work best.*/
```

Most programs running DCE-RPC in Windows 2000 or later are compiled with a specific option which will reject any request that does not demarshall correctly. Thus, is it imperative first to pass the demarshalling tests, and then to pass any application-layer tests, before finally reaching vulnerable code with a long string. s_msunistring() handles the four-byte alignment of the Unicode string automatically.

SPIKE's DCE-RPC fuzzer includes fifteen data types which it will randomly append as parameters. The intel-ordered integer values of 1,2,3 each merits their own slot in the random variable switch statement, as does one, two, and three words of zeros. `s_msunistring(s_get_random_fuzzstring());` is also used to pick a random string from the entire global set of fuzz strings and then place it in the data as a Microsoft Unicode string type. If this string is of the proper format ("/.../AAAAAA") and all the other variables are correct, and there are no extra variables, then the Locator service will process it and overflow. Finding the proper format to trigger an overflow is a matter of reading any available documentation on the service being fuzzed, and adding any interesting strings from that documentation to the global fuzz string list. The strings "/.../" and "/.:/" are often present in documentation and examples relating to the locator service. For other information about the functionality of a given RPC service, it is often only necessary to view a function's definition. In the case of the Locator service, the RpcNsStringBindingLookupBegin() function takes in roughly the arguments the vulnerability needs to demarshall.

There are 580 different strings in the SPIKE global fuzz string set. On average, once in every 170 tests, the string is actually processed, and so it will take an average of 98600 tests (or 1.5 hours), using no prior knowledge about the function's arguments (other than that "/.../AA..." or "/.:/A..." might cause an overflow), to find the Locator overflow. In fact, SPIKE was successful in finding two similar vulnerabilities within the Locator service using this methodology, and additional access violation errors not yet reported.

The time spent trying to pass the demarshalling tests can be drastically reduced by hand-coding valid arguments to the RPC function. Valid arguments can be found using standard reverse engineering, examining the exceptions thrown by the application (0x6F7 is thrown on an invalidly marshalled RPC packet) or by using Muddle, which will process an RPC Service's executable to return an IDL file. But in some cases Muddle does not work against an RPC service, and a blind approach is necessary or more expedient than a lengthy reverse engineering process.

Other Vulnerabilities Found

During the process of building the DCE-RPC over named pipe fuzzer for SPIKE 2.8, the author encountered several bugs within the Windows 2000 DCE-RPC stack. The first bug was that occasionally, when stressed, the DCE-RPC stack would forward calls to invalid interfaces or interface versions to a listening service. This would then crash the service with a NULL-dereference. In the case that the service was a necessary system service, such as lsass.exe, the system would then reboot. This is also reported as being exploitable in many circumstances as a local root against Windows 2000. When system services such as lsass.exe crash, they orphan named pipes which can then be created by a local attacker, allowing them to ImpersonateNamedPipeClient() when they are connected to by a privileged process, resulting in root compromise. It may be possible to use this technique to bypass authentication or exploit the system in other ways, although this has not yet been explored.

Additionally, a severe kernel memory leak was found when Netbios continuation packets were received by the Windows 2000 kernel. This allowed for a remote Denial of Service against a target machine. Although the machine did not itself completely run out of non-paged pool, it did stop servicing SMB requests, which has the effect of fatally disabling COM services.

Conclusion

Although static analysis or white-box analysis is the subject of much of the recent security research, black-box testing still produces the majority of the exploitable real-world vulnerabilities. For a given example security audit, SPIKE's block-based protocol modeling and fuzzing strategy has proven itself to be effective at finding exploitable vulnerabilities within a reasonable amount of time, and with a reasonable amount of prior knowledge. Software vendors and independent security consulting companies would be wise to extend SPIKE or write customized SPIKE scripts for exposed network protocols which have security implications. For a low investment of time, black-box testing, and SPIKE in particular, have consistently demonstrated themselves to be highly rewarding.

References

1. SPIKE development homepage. Http://www.immunitysec.com/spike.html
2. Muddle, a tool for reading MIDL information from binaries by Matt Chapman. Http://www.cse.unsw.edu.au/~matthewc/muddle/
3. Previous local root vulnerability against Windows 2000 based on killing lsass.exe. http://www.guninski.com/dr07.html and http://www.guninski.com/pipe3.cpp
