# Supplementary material for: Protocol vulnerability detection based on network traffic analysis and binary reverse engineering
Source: PLoS One. 2017 Oct 19;12(10):e0186188. doi: 10.1371/journal.pone.0186188 (PMC5648143; doi:10.1371/journal.pone.0186188)
Supplement: S3 File — (ZIP) [file pone.0186188.s003.zip › S3_file/TFTP programs/Tftpd32 3.51.0.0/tftpd32.351.zip!/EUPL-EN.pdf]

European Union Public Licence
v. 1.1

EUPL © the European Community 2007

This European Union Public Licence (the "EUPL") applies to the Work or Software (as defined below) which is provided under the terms of this Licence. Any use of the Work, other than as authorised under this Licence is prohibited (to the extent such use is covered by a right of the copyright holder of the Work).

The Original Work is provided under the terms of this Licence when the Licensor (as defined below) has placed the following notice immediately following the copyright notice for the Original Work:

Licensed under the EUPL V.1.1

or has expressed by any other mean his willingness to license under the EUPL.

1. Definitions

In this Licence, the following terms have the following meaning:

- *The Licence*: this Licence.

- *The Original Work* or *the Software*: the software distributed and/or communicated by the Licensor under this Licence, available as Source Code and also as Executable Code as the case may be.

- *Derivative Works*: the works or software that could be created by the Licensee, based upon the Original Work or modifications thereof. This Licence does not define the extent of modification or dependence on the Original Work required in order to classify a work as a Derivative Work; this extent is determined by copyright law applicable in the country mentioned in Article 15.

- *The Work*: the Original Work and/or its Derivative Works.

- *The Source Code*: the human-readable form of the Work which is the most convenient for people to study and modify.

- *The Executable Code:* any code which has generally been compiled and which is meant to be interpreted by a computer as a program.

- *The Licensor*: the natural or legal person that distributes and/or communicates the Work under the Licence.

- *Contributor(s):* any natural or legal person who modifies the Work under the Licence, or otherwise contributes to the creation of a Derivative Work.

- *The Licensee* or *"You":* any natural or legal person who makes any usage of the Software under the terms of the Licence.

- *Distribution* and/or *Communication*: any act of selling, giving, lending, renting, distributing, communicating, transmitting, or otherwise making available, on-line or off-line, copies of the Work or providing access to its essential functionalities at the disposal of any other natural or legal person.

2. Scope of the rights granted by the Licence

The Licensor hereby grants You a world-wide, royalty-free, non-exclusive, sub-licensable licence to do the following, for the duration of copyright vested in the Original Work:

- use the Work in any circumstance and for all usage,
- reproduce the Work,
- modify the Original Work, and make Derivative Works based upon the Work,
- communicate to the public, including the right to make available or display the Work or copies thereof to the public and perform publicly, as the case may be, the Work,
- distribute the Work or copies thereof,
- lend and rent the Work or copies thereof,
- sub-license rights in the Work or copies thereof.

Those rights can be exercised on any media, supports and formats, whether now known or later invented, as far as the applicable law permits so.

In the countries where moral rights apply, the Licensor waives his right to exercise his moral right to the extent allowed by law in order to make effective the licence of the economic rights here above listed.

The Licensor grants to the Licensee royalty-free, non exclusive usage rights to any patents held by the Licensor, to the extent necessary to make use of the rights granted on the Work under this Licence.

3. Communication of the Source Code

The Licensor may provide the Work either in its Source Code form, or as Executable Code. If the Work is provided as Executable Code, the Licensor provides in addition a machine-readable copy of the Source Code of the Work along with each copy of the Work that the Licensor distributes or indicates, in a notice following the copyright notice attached to the Work, a repository where the Source Code is easily and freely accessible for as long as the Licensor continues to distribute and/or communicate the Work.

4. Limitations on copyright

Nothing in this Licence is intended to deprive the Licensee of the benefits from any exception or limitation to the exclusive rights of the rights owners in the Original Work or Software, of the exhaustion of those rights or of other applicable limitations thereto.

5. Obligations of the Licensee

The grant of the rights mentioned above is subject to some restrictions and obligations imposed on the Licensee. Those obligations are the following:

Attribution right: the Licensee shall keep intact all copyright, patent or trademarks notices and all notices that refer to the Licence and to the disclaimer of warranties. The Licensee must include a copy of such notices and a copy of the Licence with every copy of the Work he/she distributes and/or communicates. The Licensee must cause any Derivative Work to carry prominent notices stating that the Work has been modified and the date of modification.

Copyleft clause: If the Licensee distributes and/or communicates copies of the Original Works or Derivative Works based upon the Original Work, this Distribution and/or Communication will be done under the terms of this Licence or of a later version of this Licence unless the Original Work is expressly distributed only under this version of the Licence. The Licensee (becoming Licensor) cannot offer or impose any additional terms or conditions on the Work or Derivative Work that alter or restrict the terms of the Licence.

Compatibility clause: If the Licensee Distributes and/or Communicates Derivative Works or copies thereof based upon both the Original Work and another work licensed under a Compatible Licence, this Distribution and/or Communication can be done under the terms of this Compatible Licence. For the sake of this clause, "Compatible Licence" refers to the licences listed in the appendix attached to this Licence. Should the Licensee's obligations under the Compatible Licence conflict with his/her obligations under this Licence, the obligations of the Compatible Licence shall prevail.

Provision of Source Code: When distributing and/or communicating copies of the Work, the Licensee will provide a machine-readable copy of the Source Code or indicate a repository where this Source will be easily and freely available for as long as the Licensee continues to distribute and/or communicate the Work.

Legal Protection: This Licence does not grant permission to use the trade names, trademarks, service marks, or names of the Licensor, except as required for reasonable and customary use in describing the origin of the Work and reproducing the content of the copyright notice.

6. Chain of Authorship

The original Licensor warrants that the copyright in the Original Work granted hereunder is owned by him/her or licensed to him/her and that he/she has the power and authority to grant the Licence.

Each Contributor warrants that the copyright in the modifications he/she brings to the Work are owned by him/her or licensed to him/her and that he/she has the power and authority to grant the Licence.

Each time You accept the Licence, the original Licensor and subsequent Contributors grant You a licence to their contributions to the Work, under the terms of this Licence.

7. Disclaimer of Warranty

The Work is a work in progress, which is continuously improved by numerous contributors. It is not a finished work and may therefore contain defects or "bugs" inherent to this type of software development.

For the above reason, the Work is provided under the Licence on an "as is" basis and without warranties of any kind concerning the Work, including without limitation merchantability, fitness for a particular purpose, absence of defects or errors, accuracy, non-infringement of intellectual property rights other than copyright as stated in Article 6 of this Licence.

This disclaimer of warranty is an essential part of the Licence and a condition for the grant of any rights to the Work.

8. Disclaimer of Liability

Except in the cases of wilful misconduct or damages directly caused to natural persons, the Licensor will in no event be liable for any direct or indirect, material or moral, damages of any kind, arising out of the Licence or of the use of the Work, including without limitation, damages for loss of goodwill, work stoppage, computer failure or malfunction, loss of data or any commercial damage, even if the Licensor has been advised of the possibility of such damage. However, the Licensor will be liable under statutory product liability laws as far such laws apply to the Work.

9. Additional agreements

While distributing the Original Work or Derivative Works, You may choose to conclude an additional agreement to offer, and charge a fee for, acceptance of support, warranty, indemnity, or other liability obligations and/or services consistent with this Licence. However, in accepting such obligations, You may act only on your own behalf and on your sole responsibility, not on behalf of the original Licensor or any other Contributor, and only if You agree to indemnify, defend, and hold each Contributor harmless for any liability incurred by, or claims asserted against such Contributor by the fact You have accepted any such warranty or additional liability.

10. Acceptance of the Licence

The provisions of this Licence can be accepted by clicking on an icon “I agree” placed under the bottom of a window displaying the text of this Licence or by affirming consent in any other similar way, in accordance with the rules of applicable law. Clicking on that icon indicates your clear and irrevocable acceptance of this Licence and all of its terms and conditions.

Similarly, you irrevocably accept this Licence and all of its terms and conditions by exercising any rights granted to You by Article 2 of this Licence, such as the use of the Work, the creation by You of a Derivative Work or the Distribution and/or Communication by You of the Work or copies thereof.

11. Information to the public

In case of any Distribution and/or Communication of the Work by means of electronic communication by You (for example, by offering to download the Work from a remote location) the distribution channel or media (for example, a website) must at least provide to the public the information requested by the applicable law regarding the Licensor, the Licence and the way it may be accessible, concluded, stored and reproduced by the Licensee.

12. Termination of the Licence

The Licence and the rights granted hereunder will terminate automatically upon any breach by the Licensee of the terms of the Licence.

Such a termination will not terminate the licences of any person who has received the Work from the Licensee under the Licence, provided such persons remain in full compliance with the Licence.

13. Miscellaneous

Without prejudice of Article 9 above, the Licence represents the complete agreement between the Parties as to the Work licensed hereunder.

If any provision of the Licence is invalid or unenforceable under applicable law, this will not affect the validity or enforceability of the Licence as a whole. Such provision will be construed and/or reformed so as necessary to make it valid and enforceable.

The European Commission may publish other linguistic versions and/or new versions of this Licence, so far this is required and reasonable, without reducing the scope of the rights granted by the Licence. New versions of the Licence will be published with a unique version number.

All linguistic versions of this Licence, approved by the European Commission, have identical value. Parties can take advantage of the linguistic version of their choice.

14. Jurisdiction

Any litigation resulting from the interpretation of this License, arising between the European Commission, as a Licensor, and any Licensee, will be subject to the jurisdiction of the Court of Justice of the European Communities, as laid down in article 238 of the Treaty establishing the European Community.

Any litigation arising between Parties, other than the European Commission, and resulting from the interpretation of this License, will be subject to the exclusive jurisdiction of the competent court where the Licensor resides or conducts its primary business.

15. Applicable Law

This Licence shall be governed by the law of the European Union country where the Licensor resides or has his registered office.

This licence shall be governed by the Belgian law if:

- a litigation arises between the European Commission, as a Licensor, and any Licensee;
- the Licensor, other than the European Commission, has no residence or registered office inside a European Union country.

===

Appendix

"Compatible Licences" according to article 5 EUPL are:

- *GNU General Public License (GNU GPL) v. 2*
- *Open Software License (OSL) v. 2.1, v. 3.0*
- *Common Public License v. 1.0*
- *Eclipse Public License v. 1.0*
- *Cecill v. 2.0*
